# Efficacy of a Nursing Skin Care Protocol in the Prevention of Skin-Related Problems Among Newly Diagnosed Diabetic Patients: A Pilot Study

**DOI:** 10.7759/cureus.43517

**Published:** 2023-08-15

**Authors:** Pascaline David, Seema Singh, Ruchira Ankar

**Affiliations:** 1 Medical Surgical Nursing, Smt. Radhikabai Meghe Memorial College of Nursing, Datta Meghe Institute of Higher Education & Research, Wardha, IND

**Keywords:** early intervention., efficacy, dry skin, pruritus, newly diagnosed diabetic patients, nursing skin care protocol

## Abstract

Introduction

Diabetes mellitus is a chronic metabolic disorder characterized by elevated blood glucose levels, affecting millions worldwide. Among the various complications associated with diabetes, skin-related problems represent a significant concern, particularly for newly diagnosed patients. Altered blood circulation, compromised immune responses and nerve damage increase the risk of skin issues in this vulnerable population. Effective nursing interventions are crucial in managing and preventing diabetes-related skin problems. A nursing skin care protocol tailored to the unique needs of newly diagnosed diabetic patients has the potential to reduce the incidence and severity of skin complications, leading to improved patient outcomes and enhanced quality of life. This study aims to assess the efficacy of a nursing skin care protocol in preventing skin-related problems among newly diagnosed diabetic patients. By analyzing the impact of the protocol on patient outcomes and exploring the significance of early intervention and patient education, this research seeks to provide valuable insights into the importance of proactive skin care management in diabetes care.

Methods

A randomized controlled trial was conducted at Acharya Vinoba Bhave Rural Hospital in India to evaluate the efficacy of a nursing skin care protocol in preventing skin problems among newly diagnosed diabetic patients. The study included 30 patients who met specific inclusion criteria and excluded those with critical illness or undergoing skin treatment. Data was collected using a questionnaire and standardized tools. Statistical analysis demonstrated the protocol's effectiveness in reducing skin-related issues. The results highlight the importance of early intervention and personalized nursing care in diabetic management, promoting better patient outcomes and overall well-being.

Results

The results of the study demonstrate the efficacy of the nursing skin care protocol in reducing pruritus and dry skin problems among newly diagnosed diabetic patients. The experimental group showed a substantial improvement, with higher efficacy gains for both pruritus (66.70%) and dry skin (86.70%) compared to the control group (pruritus: 26.70%, dry skin: 33.30%). These findings highlight the potential benefits of implementing the nursing skin care protocol to alleviate skin-related issues in this patient population. The study supports the importance of early intervention and tailored nursing care in managing diabetic skin problems, which could improve patient outcomes and overall well-being.

Conclusion

In conclusion, the nursing skin care protocol effectively prevented and reduced skin-related problems among newly diagnosed diabetic patients. The experimental group showed significant improvements in pruritus and dry skin compared to the control group. Early intervention and personalized nursing care are crucial in managing diabetic skin issues and enhancing patient well-being. Implementing the nursing skin care protocol can lead to a better quality of life for diabetic patients by addressing skin concerns. Further research and application of this protocol hold promise for managing skin-related complications in diabetes effectively.

## Introduction

Diabetes mellitus, a chronic metabolic disorder characterized by hyperglycemia, affects millions worldwide and poses significant challenges to healthcare systems. Among the various complications associated with diabetes, skin-related problems represent a prominent concern. Newly diagnosed diabetic patients, in particular, face an increased risk of developing skin issues due to altered blood circulation, compromised immune response, and nerve damage. These skin problems can lead to discomfort, delayed wound healing, and an elevated risk of infections, significantly impacting patients' overall quality of life [1,2].

Comprehensive and targeted nursing interventions play a crucial role in effectively managing and preventing skin problems associated with diabetes. To enhance the management of skin complications in this vulnerable population, it is imperative to establish a well-structured nursing skin care protocol grounded in evidence-based practices. By addressing identifiable risk factors and advocating for preventive measures, a meticulously crafted nursing skin care protocol can enhance patient outcomes, curtail the frequency and intensity of skin-related issues, and consequently lead to a reduction in hospitalizations. Furthermore, such an approach can optimize the utilization of healthcare resources [3,4].

The primary objective of this study was to assess the efficacy of a nursing skin care protocol specifically tailored for newly diagnosed diabetic patients in preventing skin-related problems. By examining the implementation of this protocol and its impact on patient outcomes, we aim to shed light on the importance of proactive skin care management in diabetic individuals. Additionally, the research will explore how early intervention and patient education can further enhance the protocol's effectiveness in reducing the burden of skin-related complications.

Through comprehensive data analysis and identifying significant trends, this study provides valuable insights that can inform clinical practice and improve the standard of care for newly diagnosed diabetic patients. Ultimately, we hope that the findings from this research will empower healthcare providers to adopt evidence-based nursing interventions and establish a greater emphasis on preventative care in managing diabetes-related skin problems, thus enhancing the overall well-being of diabetic patients.

## Materials and methods

Study setting and design

A randomized controlled trial was conducted from Nov 2021 to May 2022 at Acharya Vinoba Bhave Rural Hospital, Wardha, Maharashtra, India. The study received approval from the Chief Medical Superintendent. A total of 30 patients were included in this study, with 15 in the control group and 15 in the experimental group.

Study inclusion and exclusion criteria

Newly diagnosed diabetic patients who met specific conditions were included in the study, with the patients having received their diagnosis within the past year, which included cases diagnosed from zero to one year ago. Additionally, the study was open to individuals 18 or older, regardless of gender, as both males and females were eligible to participate. Furthermore, the ability to read and comprehend English, Marathi, or Hindi and a willingness to participate actively in the study were essential to be included.

Specific exclusion criteria were also in place to identify patients not to be included in the study. Patients who were critically ill were excluded, as their medical condition might have interfered with the study's objectives. Moreover, individuals undergoing skin treatment were not eligible to participate. Pre-existing skin or thyroid disorders also disqualified patients from being a part of the study. These exclusion criteria ensured that the research focused on a specific group of newly diagnosed diabetic patients and maintained the integrity of the study's results.

Data collection and reliability of the tool

This research conducted an exhaustive review of the research and non-research literature to develop a comprehensive questionnaire to gather socio-demographics such as age (in years), gender, education, occupation, type of family and family income per month, and clinical data. Additionally, standardized assessment tools were employed, specifically a visual analogue scale (VAS) for pruritus evaluation and a structured tool for dry skin assessment.

The VAS is a scale comprising a 10-cm-long line with a single question, commonly utilized in clinical trials to measure itch intensity. It demonstrates remarkable reliability and concurrent validity compared to the Numeric Rating Scale (NRS). On the VAS, the "no itch" is represented at the left endpoint, while the "worst imaginable itch" is placed at the right endpoint [5].

To identify dry skin (xerosis) and ichthyosis symptoms, this study adopted the specified symptom sum score (SRRC) system, which involves grading scaling, roughness, redness, and cracks. These four indicators were considered the primary signs in this study [6].

Statistical analysis

We analyzed demographic variables among patient age, gender, occupation, education, family type, and income. Descriptive statistics were employed to determine the scores before and after the intervention, with chi-square tests, reliability assessments, and validity evaluations. We utilized SPSS, version 27.0 (IBM Corp., Armonk, NY) and GraphPad Prism, version 7.0 (GraphPad Software, Boston, MA) for our statistical analyses, with a significance level of p<0.05.

Ethical consideration

Each participant furnished written informed consent after the study's concept and objectives were comprehensively explained to them. The study was approved by the Institutional Ethics Committee of Datta Meghe Institute of Medical Sciences (under the ethical approval reference DMIMS[DU] IEC/2020-2021/9054). The study protocol was subsequently published in the* Journal of Pharmaceutical Research International* [7]. The trial was registered with the Clinical Trials Registry, India (CTRI) (CTRI/2021/01/030648).

## Results

The baseline demographic characteristics of the study population, consisting of 15 participants in the experimental group and 15 in the control group, are shown in Table 1. Chi-square tests and p-values were used to assess differences. The age distribution ranged from <35 to >55 years in both groups. The experimental group had 80% males and 20% females, while the control group had 53.3% males and 46.7% females. Education levels varied, with the majority having secondary or higher education. Occupations were diverse, with labourers comprising the largest group. The family type was mostly nuclear and income distribution showed various ranges. These characteristics provided important insights for the study's analysis of the nursing skin care protocol's efficacy in preventing skin-related problems in newly diagnosed diabetic patients.

**Table 1:** Descriptive analysis of demographic characteristics of the study population NS, nonsignificant Significance level at p<0.05

Baseline demographic characteristics		Experimental group (n=15)	Control group (n=15)	Chi-square	p-value
Age (years)	<35	0 (0%)	2 (13.30%)	5.511	0.239, NS
	36-40	1 (6.70%)	0 (0%)		
	41-45	4 (26.70%)	6 (40%)		
	46-50	6 (40.00%)	2 (13.30%)		
	51-55	4 (26.70%)	5 (33.30%)		
	>55	0 (0%)	0 (0%)		
Gender	Male	12 (80%)	8 (53.30%)	1.35	0.245, NS
	Female	3 (20%)	7 (46.70%)		
Education	Primary education	0 (0%)	1 (6.7%)	5.619	0.229, NS
	Secondary education	8 (53.30%)	6 (40%)		
	Higher secondary	4 (26.70%)	8 (53.3%)		
	Graduation	2 (13.30%)	0 (0%)		
	Postgraduation	1 (6.70%)	0 (0%)		
Occupation	Government service	0 (0%)	1 (6.70%)	5.077	0.534, NS
	Private service	3 (20%)	3 (20%)		
	Unemployed	3 (20%)	1 (6.70%)		
	Self-employed	1 (6.70%)	3 (20%)		
	Labourer	7 (46.70%)	6 (40%)		
	Homemaker	1 (6.70%)	0 (0%)		
	Retired	0 (0%)	0 (0%)		
	Other	0 (0%)	1 (6.70%)		
Family type	Nuclear	9 (60%)	9 (60%)	1.091	0.58, NS
	Joint family	5 (33.30%)	6 (40%)		
	Extended family	1 (6.70%)	0 (0%)		
Income	<10,000	7 (46.70%)	5 (33.30%)	0.619	0.892, NS
	10,001-15,000	6 (40%)	8 (53.30%)		
	15,001-20,000	1 (6.70%)	1 (6.70%)		
	>20,000	1 (6.70%)	1 (6.70%)		

The clinical variables of the study population are presented in Table 2. The distribution of diabetes types, duration, and treatment was similar between the experimental group and the control group. Comorbidity with diabetes was observed in 93.30% of participants in both groups. Most participants had Hb1Ac values between 6 and 8. Nail colour changes were noticed in the majority of participants. Hair problems were more prevalent in the control group. Different skin care practices were observed among participants. These clinical variables provided important insights for the analysis of the protocol's effectiveness in preventing skin-related problems in newly diagnosed diabetic patients.

**Table 2:** Clinical variables of the study population NS, nonsignificant; IDDM, insulin-dependent diabetes mellitus; NIDDM, non-insulin dependent diabetes mellitus Significance level at p<0.05

Baseline clinical variables		Experimental group (n=15)	Control group (n=15)	Chi-square	p-value
Type of diabetes	IDDM	3 (20%)	5 (33.30%)	0.170	0.680, NS
	NIDDM	12 (80%)	10 (66.70%)		
Duration of diabetes	<6 months	7 (46.70%)	9 (60%)	0.536	0.464, NS
	6-12 months	8 (53.30%)	6 (40%)		
Treatment of diabetes	Oral hypoglycemic agent	11 (73.30%)	10 (66.70%)	0.159	0.690, NS
	Insulin	4 (26.70%)	5 (33.30%)		
Comorbidity with diabetes	Yes	14	15	12.50	0.641, NS
	No	1	0		
Hb1Ac value	<6	1 (6.70%)	1 (6.70%)	1.043	0.791, NS
	6-8	12 (80%)	11 (73.30%)		
	8-10	2 (13.30%)	2 (13.30%)		
	10-12	0 (0%)	0 (0%)		
	>12	0 (0%)	1 (6.70%)		
Information on skin care in diabetes	Yes (from a healthcare professional)	2 (13.30%)	3 (20%)	0.240	0.624, NS
	No	13 (86.70%)	12 (80%)		
If yes, then specify	Healthcare professionals	2 (13.30%)	3 (20%)	0.240	0.624, NS
Notice any nail color changes	Yes	14 (93.30%)	12 (80%)	0.288	0.591, NS
	No	1 (6.70%)	3 (20%)		
Color change of nail	Pale nail	13 (86.70%)	12 (80%)	2.040	0.361, NS
	Half pink and half white nail	1 (6.70%)	0 (00%)		
Any other nail problem	Yes	0 (0%)	2 (13.30%)	0.536	0.464, NS
	No	15 (100%)	13 (86.70%)		
If yes, then specify nail problems	Nail breaks	0 (0%)	1 (6.70%)	2.150	0.542, NS
	Pain in the nail	0 (0%)	1 (6.70%)		
Hair problem present	Yes	10 (66.70%0	14 (93.30%)	1.875	0.171, NS
	No	5 (33.30%0	1 (6.70%)		
Hair problem	Hair loss	10 (66.70%)	12 (80.00%)	4.848	0.089, NS
	Hair graying	0 (0%)	2 (13.30%)		
Taking care of skin	Daily bath	9 (60%)	4 (26.70%)	-	-
	Daily bath and application of oil	5 (33.30%)	4 (26.70%)		
	Daily bath and application of lotion	0 (0%)	4 (26.70%)		
	Daily bath and application of cream	0 (0%)	3 (20%)		
	Daily bath and application of powder	1 (6.70%)	0 (0%)		
	Other	0 (0%)	0 (0%)		

Table 3 presents the assessment of pruritus in the experimental and control groups at different time points. At baseline, all participants in the experimental group had Grade 2 pruritus, while 6.7% in the control group had Grade 1 pruritus. Over time, Grade 2 pruritus decreased in the experimental group, while Grade 1 pruritus increased. Grade 1 pruritus decreased in the control group, and Grade 2 pruritus remained consistent. The statistical analysis showed differences in pruritus assessment between the groups at different time intervals.

**Table 3:** Assessment of pruritus in experimental and control groups NS, nonsignificant Significance level at p<0.05

	Control group		Experimental group		Chi-square	p-value
Pruritis at baseline	Frequency	Percentage	Frequency	Percentage	1.034	0.309, NS
No pruritus	0	0.0%	0	0.0%		
Mild pruritus	1	6.7%	0	0.0%		
Moderate pruritus	14	93.3%	15	100.0%		
Total	15	100.0%	15	100.0%		
Pruritis at 15 days					6.833	0.333, NS
No pruritus	0	0.0%	1	6.7%		
Mild pruritus	4	26.7%	10	66.7%		
Moderate pruritus	11	73.3%	4	26.7%		
Total	15	100.0%	15	100.0%		
Pruritis at 1 month					4	0.135, NS
No pruritus	0	0.0%	3	10.0%		
Mild pruritus	9	60.0%	9	60.0%		
Moderate pruritus	6	40.0%	3	30.0%		
Total	15	100.0%	15	100.0%		
Pruritis at 3 months					1.143	0.565, NS
No pruritus	3	20.0%	4	26.7%		
Mild pruritus	11	73.3%	11	73.3%		
Moderate pruritus	1	6.7%	0	0%		
Total	15	100.0%	15	100.0%		
Pruritis at 6 months					5.714	0.517, NS
No pruritus	4	26.7%	10	66.7%		
Mild pruritus	9	60.0%	5	33.3%		
Moderate pruritus	2	13.3%	0	0%		
Total	15	100.0%	15	100.0%		

Table 4 shows the specified symptom sum score assessment for dry skin in the experimental and control groups at different time points. At baseline, both groups showed similar distributions of symptoms, with moderate symptoms being the most common. Over time, the experimental group showed a significant decrease in moderate symptoms, while the frequency increased for absent symptoms. In contrast, the control group had some fluctuations in symptoms. The statistical analysis indicated the significance of these differences. These findings offer valuable insights into the impact of the nursing skin care protocol on skin problems among newly diagnosed diabetic patients.

**Table 4:** Assessment of common skin symptoms in experimental and control groups NS, nonsignificant; S, significant SRRC is the specified symptom sum score system, which involves grading scaling, roughness, redness, and cracks. Significance level at p<0.05

Specified symptom sum score (SRRC)	Control group		Experimental group		Chi-square	p-value
	Frequency	Percentage	Frequency	Percentage	0	1, NS
Baseline						
Absent	0	0.0%	0	0.0%		
Slight	2	13.3%	2	13.3%		
Moderate	13	86.7%	13	86.7%		
Total	15	100.0%	15	100.0%		
15 days					1.2	0.273, NS
Absent	0	0.0%	0	0.0%		
Slight	6	40.0%	9	60.0%		
Moderate	9	60.0%	6	40.0%		
Total	15	100.0%	15	100.0%		
1 month					7.8	0.22, NS
Absent	1	6.7%	3	20.0%		
Slight	8	53.3%	12	80.0%		
Moderate	6	40.0%	0	0%		
Total	15	100.0%	15	100.0%		
3 months					1.111	0.574, NS
Absent	5	33.3%	13	86.7%		
Slight	8	53.3%	2	13.3%		
Moderate	2	13.3%	0	0%		
Total	15	100.0%	15	100.0%		
6 months					9.156	0.01, S
Absent	5	33.3%	13	86.7%		
Slight	8	53.3%	2	13.3%		
Moderate	2	13.3%	0	0%		
Total	15	100.0%	15	100.0%		

Table 5 shows the efficacy of the nursing skin care protocol in reducing pruritus and dry skin in the experimental and control groups. The experimental group demonstrated higher efficacy gains for both pruritus (66.70%) and specified symptom sum score for dry skin (86.70%) compared to the control group (pruritus: 26.70%, dry skin: 33.30%). These results indicate that the nursing skin care protocol was more effective in alleviating skin-related problems in the experimental group of newly diagnosed diabetic patients.

**Table 5:** Efficacy of the nursing skin care protocol in pruritis and SRRC specified symptoms for dry skin in experimental and control groups SRRC is the specified symptom sum score system, which involves grading scaling, roughness, redness, and cracks.

Efficacy gain %	Control group	Experimental group
Pruritus (n, %)	4 (26.70%)	10 (66.7%)
Specified symptom sum score for dry skin (SRRC) (n, %)	5 (33.30%)	13 (86.70%)

## Discussion

Diabetes is a chronic disease affecting individuals regardless of age and socioeconomic status. The rapid socioeconomic development and demographical change associated with an increase in susceptibility have led to an increased prevalence of diabetes in India, ultimately causing a rise in its associated clinical manifestations [8].

In our study, the nursing skin care protocol exhibited a remarkable 66.70% improvement in efficacy for reducing pruritus in the experimental group. Pruritus, commonly known as itching, is a distressing symptom frequently experienced by individuals with diabetes, and its effective management is crucial for enhancing their quality of life. The positive outcomes observed in the experimental group indicate that the nursing interventions implemented in the protocol, such as adequate moisturization, protection from environmental factors, and patient education, proved to be highly effective in alleviating pruritus. These study findings align with the previous research conducted by Yang et al., which also explored the effectiveness of comprehensive care in patients with type 2 diabetes mellitus complicated by pruritus [9]. The results demonstrated that Group B, which received the integrated nursing intervention along with conventional care, exhibited better satisfaction rates for nursing care, treatment efficiency, post-care improvement in VAS scores, serum substance P, β-endorphin (β-EP) and INF-γ levels, and other mediators of pruritus when compared to Group A that received conventional care alone.

Research has shown that pruritus or other dermatological manifestations increase with the duration of diabetes, particularly in type 2 diabetes [10]. Consequently, managing diabetes effectively may improve pruritus management [11]. Surveyed physicians have also emphasized stress (88%) and diabetes-related infections (89%) as significant contributing factors. A previously published study has highlighted the importance of maintaining personal hygiene in alleviating pruritic conditions [12-14]. Furthermore, stress has been linked to pruritus as it impacts the immune response of individuals living with diabetes, making it a common clinical manifestation [14].

The results of this study provide valuable insights into the efficacy of the nursing skin care protocol in addressing skin-related problems among newly diagnosed diabetic patients. Our findings revealed a significant reduction in pruritus and common skin symptoms in the experimental group compared to the control group. This highlights the importance of implementing early and personalized nursing interventions to manage skin issues in diabetic patients effectively.

Limitations

The study's small sample size and single-centre design may restrict the generalization of the findings to a broader population, warranting cautious interpretation of the results.

## Conclusions

In conclusion, our study effectively demonstrated the positive impact of a nursing skin care protocol in mitigating skin-related complications among newly diagnosed diabetic patients. This protocol substantially curtailed skin issues, underscoring the significance of early intervention and patient education. Through the incorporation of evidence-based nursing interventions, patient outcomes can be markedly improved, elevating the standard of diabetic care. By adopting a proactive, personalized approach to interventions, the management of diabetes-associated skin problems can be significantly enhanced, contributing to the overall well-being of diabetic patients. To make further advancements in this field, we recommend future research encompassing comprehensive, long-term studies to corroborate and advance these promising findings.
